# Multidisciplinary recommendations for palliative and supportive care in Creutzfeldt-Jakob disease and related disorders

**DOI:** 10.1093/ageing/afag206

**Published:** 2026-07-19

**Authors:** Melissa Ng, Anna Grundy, Brian Appleby, Terri Awe, Matthew Carey, Edgar Chan, Chloe Chin, Ruth Diver, Peter Hermann, Alexsandra Kovacevich, Nuriye Kupeli, Ann Rochelle Marasigan, Beth Marsh, Jonathan Martin, Kosuke Matsuzono, Eugene Ace McDermott, Kirsty McNiven, Mary Miller, Lucy Pauli, Rachel Quibell, Diane Ritchie, Alex Ruck Keene, Elizabeth L Sampson, George Turner, Rachel Williams, Masahito Yamada, Inga Zerr, Tomasz Bajorek, Kristiam Doughty Herrera-Carrasco, James Kennard, Veronica O'Donnell, Richard Knight, John Collinge, Simon Mead, Victoria Bradley

**Affiliations:** National Prion Clinic, National Hospital for Neurology and Neurosurgery, University College London Hospitals NHS Foundation Trust, London, UK; Department of Palliative Care, Newcastle Upon Tyne Hospitals NHS Foundation Trust, Newcastle Upon Tyne, UK; National Prion Disease Pathology Surveillance Center, Case Western Reserve University School of Medicine, Cleveland, OH, USA; Department of Neurology, Case Western Reserve University School of Medicine, Cleveland, OH, USA; Department of Psychiatry, Case Western Reserve University School of Medicine, Cleveland, OH, USA; National CJD NHS Nursing Service, NHS Lothian, Edinburgh, UK; Department of Palliative Care, Oxford University Hospitals NHS Foundation Trust, Oxford, UK; Department of Neuropsychology, National Hospital for Neurology and Neurosurgery, London, UK; Department of Palliative Care, Cambridge University Hospitals NHS Foundation Trust, Cambridge, UK; Department of Palliative Care, Cambridge University Hospitals NHS Foundation Trust, Cambridge, UK; National Reference Center for Surveillance of TSE, University Medical Center Göttingen, Göttingen, Germany; Department of Psychiatry, Case Western Reserve University School of Medicine, Cleveland, OH, USA; Marie Curie Palliative Care Research Department, University College London, London, UK; Wolfson Institute of Population Health—Centre for Psychiatry and Mental Health, Queen Mary University of London, London, UK; National Prion Clinic, National Hospital for Neurology and Neurosurgery, University College London Hospitals NHS Foundation Trust, London, UK; CJD Support Network, Chester, UK; Department of Palliative Care, National Hospital for Neurology and Neurosurgery, London, UK; MRC Prion Unit at UCL, Institute of Prion Diseases, London, UK; Department of Neurology, Jichi Medical University, Tochigi, Japan; Salisbury NHS Foundation Trust, Salisbury, UK; National Prion Clinic, National Hospital for Neurology and Neurosurgery, University College London Hospitals NHS Foundation Trust, London, UK; Department of Palliative Care, Oxford University Hospitals NHS Foundation Trust, Oxford, UK; Department of Psychiatry, Rotherham Doncaster and South Humber NHS Foundation Trust, Doncaster, UK; Department of Palliative Care, Newcastle Upon Tyne Hospitals NHS Foundation Trust, Newcastle Upon Tyne, UK; Centre for Clinical Brain Sciences, University of Edinburgh Division of Clinical and Surgical Sciences, Edinburgh, UK; 39 Essex Chambers, London, UK; Marie Curie Palliative Care Research Department, University College London, London, UK; Wolfson Institute of Population Health—Centre for Psychiatry and Mental Health, Queen Mary University of London, London, UK; Marie Curie Palliative Care Research Department, University College London, London, UK; National Prion Clinic, National Hospital for Neurology and Neurosurgery, University College London Hospitals NHS Foundation Trust, London, UK; Department of Neurology, Kudanzaka Hospital, Tokyo, Japan; Department of Neurology and Neurological Science, Institute of Science Tokyo, Tokyo, Japan; Department of Neuropsychology, National Hospital for Neurology and Neurosurgery, London, UK; German Centre for Neurodegenerative Diseases, Göttingen, Germany; Neuropsychiatry, Oxford University Hospitals NHS Foundation Trust, Oxford, UK; Marie Curie Palliative Care Research Department, University College London, London, UK; Banbury Cross Health Centre, Banbury, UK; National Prion Clinic, National Hospital for Neurology and Neurosurgery, University College London Hospitals NHS Foundation Trust, London, UK; National CJD Diagnostic and Advisory Service, Edinburgh, UK; National Prion Clinic, National Hospital for Neurology and Neurosurgery, University College London Hospitals NHS Foundation Trust, London, UK; MRC Prion Unit at UCL, Institute of Prion Diseases, London, UK; National Prion Clinic, National Hospital for Neurology and Neurosurgery, University College London Hospitals NHS Foundation Trust, London, UK; MRC Prion Unit at UCL, Institute of Prion Diseases, London, UK; Department of Palliative Care, Oxford University Hospitals NHS Foundation Trust, Oxford, UK

**Keywords:** Creutzfeldt-Jakob disease, prion disease, rapidly progressive dementia, older people

## Abstract

Prion diseases, of which Creutzfeldt-Jakob disease is the most common, are fatal neurodegenerative disorders and are often rapidly progressive. They are associated with a significant palliative care burden for patients and families, ranging from prognostic uncertainty to complex symptom management to caregiver distress. Healthcare professionals face unique pressures when caring for these patients, which can include a lack of familiarity with this rare diagnosis and rapidly evolving symptom needs due to accelerated clinical deterioration. We convened a multidisciplinary panel of experts from around the UK, including palliative care doctors, general practitioners, physician and nurse specialists in prion diseases, and a lived experience representative to compile practical, consensus-based recommendations for managing prion diseases, much of which can also be applied to other rapidly progressive dementias. In this article, we examine the available evidence base for managing various aspects of prion diseases. Where evidence is limited, we suggest best practices informed by decades of our collective experiences.

## Key Points

- Multidisciplinary involvement is essential in the care of patients with prion diseases.
- Supportive and palliative care of patients with prion diseases is particularly challenging due to the rapid clinical course.
- Knowledge of local and national resources is helpful to support patients and professionals dealing with prion diseases.
- Due to the rarity of the condition, the evidence base for symptomatic management of prion diseases is very limited.

## Introduction and background to prion diseases

### Aetiology and epidemiology

Prion diseases are a group of rare and incurable neurodegenerative disorders of mammals associated with the accumulation of abnormally folded prion protein. There are no known cures or disease-modifying treatments; the mainstay of treatment is supportive care and symptom management. Creutzfeldt-Jakob disease (CJD) is the most common prion disease in humans. Most cases (85%) are sporadic, meaning the cause is unknown, although the naturally occurring polymorphism at codon 129 of the prion protein gene (*PRNP*) affects genetic susceptibility [1]. Sporadic CJD has a median annual mortality rate of 1.46 per million in the UK [2].

Inherited prion disease (IPD), including familial or genetic CJD, Gerstmann-Sträussler-Scheinker disease, Fatal Familial Insomnia and a spectrum of other phenotypes, is associated with mutations or insertions in the *PRNP* gene and accounts for around 10%–15% of cases. IPD is inherited in an autosomal dominant pattern, meaning that first-degree relatives of an affected person are each at 50% risk of also carrying the gene mutation.

Rarely (<1% of cases), prion disease is acquired through contaminated human (iatrogenic transmission through certain medical interventions) or bovine (ingestion of beef infected with Bovine Spongiform Encephalopathy, resulting in variant CJD) sources. Most iatrogenic cases are due to the administration of contaminated cadaveric-derived growth hormone or contaminated cadaveric-derived dural grafts, which are no longer used since the advent of synthetic substitutes. A very small number of iatrogenic cases are linked to neurosurgical procedures using contaminated instruments [3]. The variant CJD epidemic reached its peak in the late 1990s/early 2000s, with the last known UK case reported in 2016 [4].

### Clinical features

**Figure 1:**
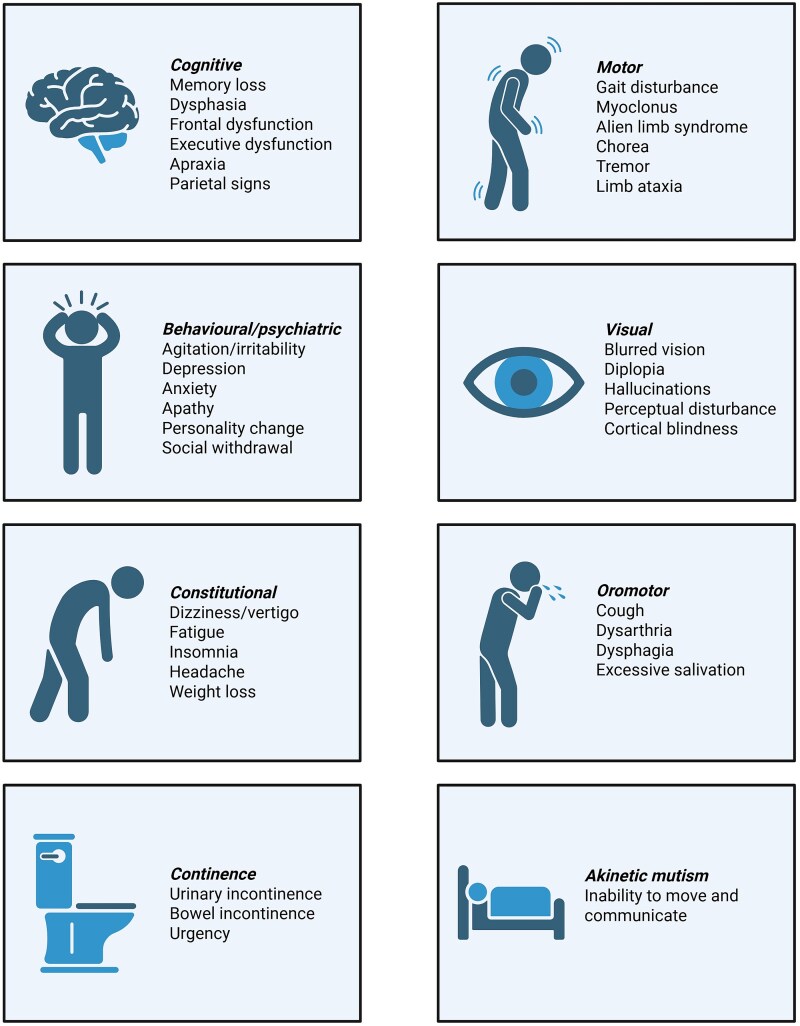
Clinical features of prion diseases. *Created in BioRender. One, S. (2025) https://BioRender.com/pfwsyth*.

The onset of sporadic CJD typically occurs between the ages of 50–80 years. In IPD, there is variation in the age of onset, in part depending on the *PRNP* mutation. IPD symptoms are most likely to develop between the ages of 30–75. Prion diseases are highly heterogeneous in terms of presentation and progression in the early part of the disease, even within aetiological groups. Figure 1 shows the range of symptoms seen in prion diseases. The clinical syndromes at presentation include (from most to least common) [5]:

- Classical (50%): rapidly progressive cognitive decline, cerebellar ataxia, myoclonus and pyramidal/extrapyramidal signs
- Pure cognitive (15%): no significant motor features
- Pure ataxic (10%): no significant cognitive involvement
- Psychiatric/behavioural (5%): mood disorder, personality change, paranoia, visual hallucinations and aggression
- Pure visual, also known as the Heidenhain variant (5%): cortical visual impairment, visual hallucinations and rapidly progressive course
- Sleep/thalamic (2%): sleep disorder, peripheral pain and sensory disturbance
- Stroke-like (2%): rapid-onset and asymmetrical motor symptoms
- Corticobasal (2%): asymmetric extrapyramidal signs and alien limb syndrome

As the illness progresses, there is continued functional decline. Some symptoms, for example behavioural and psychiatric symptoms and hyperkinetic movement disorders such as chorea or alien limb syndrome, fluctuate and may even improve or resolve spontaneously over time [6, 7]. This potentially occurs because of rapidly progressive global neurological disability which renders patients less able to manifest these symptoms. Other symptoms, such as increased tone and myoclonus, worsen with disease progression. The disease becomes increasingly homogeneous towards the advanced stages of akinetic mutism (a state of being unable to move or communicate) and end of life.

### Diagnosis

There are standard epidemiological diagnostic criteria for definite, probable and possible CJD [8]. Definite diagnosis requires neuropathological, immunocytochemical or biochemical confirmation of prion disease in brain tissue, usually obtained post-mortem or via brain biopsy, although this is now rarely required. Probable diagnosis is based on clinical features (rapidly progressive dementia, myoclonus, visual or cerebellar symptoms, pyramidal or extrapyramidal features and akinetic mutism), and investigations (Real-Time Quaking-Induced Conversion (RT-QuIC) assay in cerebrospinal fluid or other tissues, high signal in caudate/putamen on MRI or at least two cortical regions either on diffusion-weighted imaging or fluid attenuated inversion recovery, electroencephalogram demonstrating periodic sharp wave complexes). A diagnosis of iatrogenic CJD is made in the context of a recognised exposure risk. To meet the criteria for IPD, there must be a progressive neuropsychiatric disorder with either a supportive family history or a disease-specific *PRNP* gene mutation.

### Prognosis

Median survival in sporadic CJD is ~4–6 months [9], with the large majority of people dying within 1 year of symptom onset. Several factors are associated with a poorer prognosis, including methionine homozygosity at codon 129, older age at onset and male sex [10]. Duration of illness in IPD varies widely depending on the mutation, ranging from several weeks to many years.

**Figure 2:**
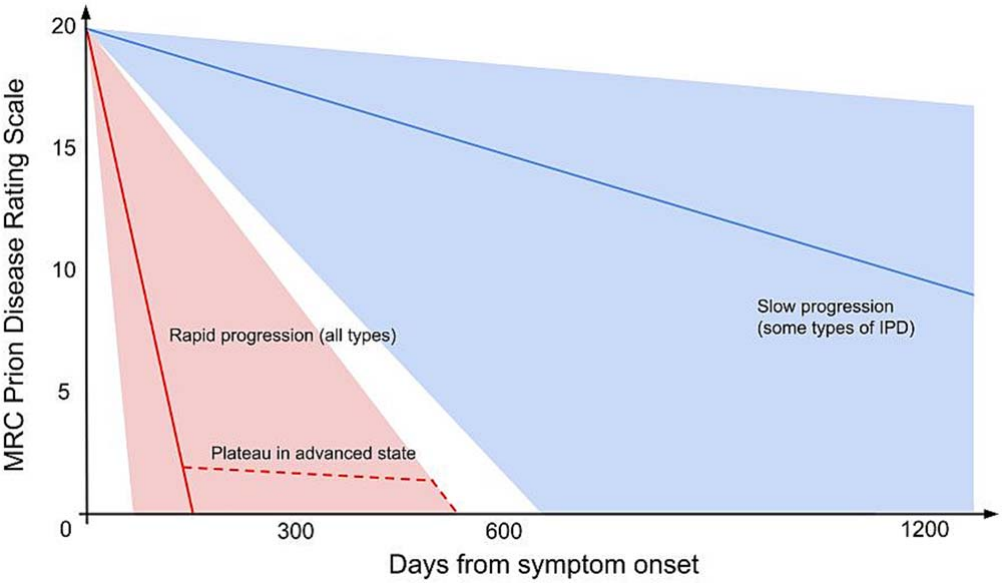
Patterns of progression seen in prion diseases. The shaded areas are illustrative ranges seen within the trajectories. Adapted from Thompson *et al.*, 2013 [12].

The Medical Research Council Prion Disease Rating Scale (MRC Scale) is a validated 20-point tool that captures functional decline over time and reflects disease progression [11]. A score of 20 corresponds to a person who may have symptoms but no disability, whereas low scores correspond to advanced states of neuro-disability such as akinetic mutism. Longitudinal use of this scale identified three common trajectories: a rapid, generally linear decline over weeks to months (seen across all aetiological groups); a rapid decline followed by a prolonged plateau in a state of severe neuro-disability; and a more gradual decline over several years (seen almost exclusively in IPD) [12] (see Figure 2).

Families should be supported to understand that akinetic mutism marks the advanced stage of prion disease, but survival in this phase can vary widely and unpredictably. For example, survival may be prolonged by clinically-assisted nutrition and hydration (CANH), or shortened by complications, such as the development of an aspiration pneumonia. Understanding the priorities of the person and family is important to ensure goal-concordant care. Communicating prognostic information with sensitivity is essential, acknowledging both the typical disease course and the inherent uncertainty in individual cases.

## Objectives

Due to the rarity of the conditions and the rapidity of clinical deterioration, the available evidence base relating to the care of prion diseases is limited, comprising observational cohort studies, case reports, case series and expert opinion. There are no randomised controlled trials related to the symptomatic management of prion diseases. The objective of this guideline is to provide comprehensive and pragmatic recommendations informing the palliative care and symptom management of people with prion diseases. This guideline is intended for healthcare professionals involved in the care of people with prion diseases. It may also be of interest to patients and families facing prion diseases to advocate for high quality, patient-focused care and to learn about local and national levels of support available.

## Method

We hosted a hybrid on-site and remote workshop comprising multidisciplinary specialists on prion diseases from around the UK. The panel consisted of physician, nurse and research specialists in prion diseases, palliative care doctors, psychiatrists, a general practitioner, a clinical neuropsychologist and a lived experience representative. The initial workshop served to assess the landscape of available evidence, decide which symptom clusters to prioritise, and suggest expert recommendations. Individual authors with particular interest or experience in an area then reviewed the relevant literature and drafted the subsections. The development of this guideline followed, with iterative collaboration among the authors to refine recommendations. The involvement of a lived experience representative was integral to ensure recommendations were patient-focused, and we included a section on support for families and professionals, which focused on the impact on and resources for caregivers.

A literature search of PubMed was conducted on 26 January 2026 using a search strategy targeted at identifying articles addressing symptomatic management of prion diseases. Papers were included if they addressed human participants with a diagnosis of prion disease and focused on symptom management. Any comparative intervention and study design was considered, and the papers had to describe the impact on symptoms. Papers were excluded if they were non-human studies, non-prion studies, focused only on diagnostic findings or genetic associations, focused only on disease-modifying treatments or single case reports without addressing symptom management. The resulting papers were reviewed by an author and referenced in the manuscript where appropriate.

A subsequent hybrid workshop was held where the final manuscript was reviewed and agreed upon, and future actions were identified. International prion disease specialists were invited to review the manuscript and comment on practice differences in Japan, Germany and the United States of America. The panel will reconvene in 5 years to reflect on learning in clinical practice, examine new evidence and update recommendations.

## Multidisciplinary collaboration

**Figure 3:**
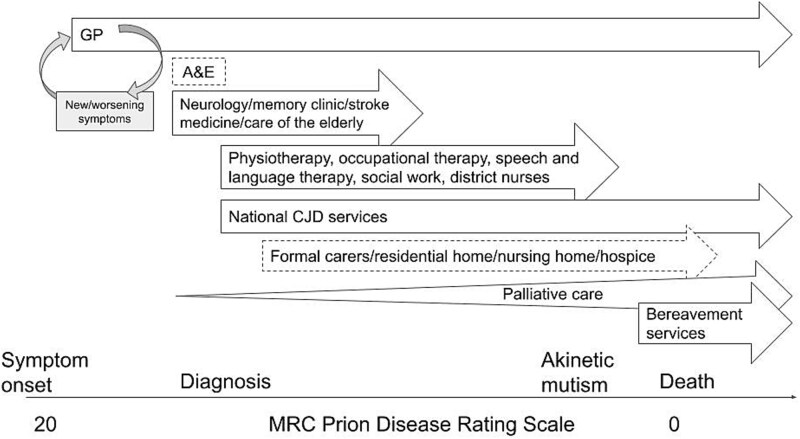
Multidisciplinary involvement throughout the disease trajectory in prion diseases. Note there is significant variation, indicated by the dashed lines, and for example, some people are diagnosed in very advanced stages and may die in hospital without significant involvement from other services.

Collaborative working between different healthcare professionals is vital to provide individualised care which addresses the diverse and rapidly changing needs of people with prion diseases [13]. Figure 3 illustrates the involvement of multidisciplinary team (MDT) members throughout the disease trajectory. Interventions should aim to support function and maintain dignity for as long as possible [14]. See Supplementary Appendix S1 for a demonstrative case study outlining some of the teams and challenges associated with caring for people with prion diseases.

We recommend the involvement of:

- **General practitioners** (a.k.a. primary care or family doctors) to provide continuity of care and support not only to the individual but also to the wider family for their associated needs during a physically and psychologically demanding time.
- **Occupational therapists** to assess the home for adaptations and provide specialist equipment.
- **Physiotherapists** to assist with gait and balance training in cases of early mobility impairment.
- **Speech and language therapists** to assess and manage communication and swallowing impairment.
- **District nurses** (a.k.a. visiting, community or home health nurses) for management of skin and continence issues and provision of end-of-life care.
- **Geriatricians and old age psychiatrists** to help manage any complex chronic conditions and challenging neuropsychiatric symptoms.
- **Palliative care** for advance or future care planning (ideally while the person is still able to contribute to these discussions [15]), symptom management and end-of-life care [16].
- **Social work** for care planning and, in the UK, assessing eligibility for free NHS-funded health and social care. Additional funding for care can be sought from the National CJD Care Fund and the CJD Support Network Care Grants [17].
- **Specialists** from the National Prion Clinic and the National CJD NHS Nursing Service (see Table 1). All individuals with suspected prion disease in the UK should be referred to the National Prion Clinic. The level of specialist support outside the UK varies. Local resources can be found via the CJD International Support Alliance (see Table 1).

Regular communication to share updates and adjust care plans as needed is key for collaborative working among the MDT. Patients and families should be involved in care decisions to ensure that treatment plans reflect their preferences and values. National CJD services play an essential role in providing education and training for local services so that MDT members can stay updated on best practices in care of people with prion diseases.

## Future care planning

Future care planning (FCP) is a voluntary process in which individuals and their families discuss and record their preferences for future care, giving them reassurance that their wishes will be respected when they lose capacity. FCP results in increases in people receiving care consistent with their goals, reduced hospital utilisation and increased patient satisfaction with clinicians and medical care [18]. It may involve appointing a proxy or power of attorney, making advance decisions to refuse specific treatments (such as CANH or intravenous antibiotics), completing advance statements about personal values and care preferences and agreeing treatment escalation plans. FCP is also known as advance care planning in some countries, although advance care planning requires the person to have decision-making capacity, whereas FCP includes making decisions in the best interests of people who lack capacity [19].

**Table 1:** Directory of resources to support families and professionals dealing with prion diseases. Details correct at time of publication. Up-to-date information is available at https://www.ucl.ac.uk/national-prion-clinic

Organisation	Link	Description
**Care and Support Statutory Guidance**	https://www.gov.uk/government/publications/care-act-statutory-guidance/care-and-support-statutory-guidance	Support for local authorities in England to implement the Care Act 2014. Paragraph 8.14 pertains to care and support for people with CJD.
**Child Bereavement UK**	https://www.childbereavementuk.org	UK charity supporting children and families to rebuild their lives when a child grieves or when a child dies. They support children and young people up to the age of 25.
**Citizens Advice Bureau**	https://www.citizensadvice.org.uk/	UK national charity and network of local charities offering guidance on a wide range of issues, including financial and legal issues.
**CJD Guidelines for Social Workers**	https://www.gov.uk/government/publications/creutzfeldt-jakob-disease-cjd-guidelines-for-social-workers	CJD guidelines for Social Workers published in June 2018.
**CJD International Support Alliance**	https://cjdisa.com/	International coalition nonprofit organisations working on behalf of those affected by or at risk of prion disease.
**CJD Support Network**	https://www.cjdsupport.co.uk	UK charity offering information and support and connecting those affected by and at risk of all forms of prion disease.
**Cruse Bereavement Support**	https://www.cruse.org.uk	UK’s leading bereavement charity, offering support through their website, national helpline, live chat, group or one-to-one in-person and online support.
**National CJD Nursing Service**	https://services.nhslothian.scot/cjds/	National specialist nursing service dedicated to supporting people diagnosed with CJD and their families.
**National Prion Clinic**	https://www.ucl.ac.uk/national-prion-clinic	National referral centre for prion disease in the UK that is part of the University College London Hospitals NHS Foundation Trust (UCLH).
**NHS**	https://www.nhs.uk/conditions/creutzfeldt-jakob-disease-cjd/	Information about CJD on the NHS website.
**Rare Minds Wellbeing Hub**	https://www.rareminds.org/wellbeing-hub/	Resource hub to support the mental health of those with rare conditions.
**Samaritans**	https://www.samaritans.org	UK charity that provides confidential emotional support to people who are struggling with their feelings, offering a non-judgmental listening service 24/7.
**vCJD Trust**	https://www.vcjdtrust.co.uk	Information about the compensation scheme for victims of variant CJD and their families.
**WAY (Widowed and Young)**	https://www.widowedandyoung.org.uk/	UK peer-to-peer support charity for people aged 50 or under when their partner died (regardless of marital status, gender or sexual orientation).
**Way Up**	https://way-up.co.uk/	Peer-to-peer support group for those widowed primarily, but not exclusively, in their 50s and 60s.

People with prion diseases face an accelerated timeline and therefore opportunities to allow them to express their preferences and priorities should be taken as soon as possible, ideally at the time of diagnosis. This can be particularly challenging as it is often a period of high emotional stress, and during the time taken to reach diagnosis, the person may have lost decision-making capacity. Early involvement of palliative care is encouraged to help guide these conversations.

Where no family or friends are available to consult in a best interests decision-making process, an advocate or representative, such as an Independent Mental Capacity Advocate in the UK, can be appointed according to the local legal framework to support decision-making and ensure that due process is followed [20]. In the UK, clinicians may complete a Special Rules for end of life (SR1) form to enable rapid access to benefits [21], and liaise with the general practitioner to add the patient to the Gold Standards Framework register to support coordinated palliative care [22].

## Support for families and professionals

Caring for a loved one in the process of dying is often a profoundly emotional experience. We present here some things to consider in the context of prion diseases and Table 1 provides a list of resources that may be helpful.

### Diagnostic process and interaction with clinicians

The journey to diagnosis is often marked by uncertainty, misdiagnosis and repeated cycles of hope and loss [23]. Lack of disease-specific knowledge among professionals often compounds feelings of frustration and mistrust [24–26]. It is important that professionals recognise this context and invest in building trust with families, ensuring that they are well informed through close liaison with specialist clinicians [27]. Clear and compassionate communication is particularly paramount. Table 2 shows some suggested answers to commonly asked questions.

**Table 2:** Suggested answers to commonly asked questions about prion diseases

What is Creutzfeldt-Jakob disease?
CJD is the most common form of prion disease in humans. It is a serious illness of the brain, and it is always fatal. In CJD, a normal brain protein, called the prion protein, folds into the wrong shape. These misfolded proteins can build up, spread through the brain and cause damage. For now, there is no treatment that can slow down or cure CJD. Care focuses on relieving symptoms, helping the person and their family, and keeping the person as calm and comfortable as possible.
How does someone get CJD?
There are different types of CJD, and they happen in different ways. The most common type is called sporadic CJD. In this type, the prion protein changes shape by accident. We do not yet know why this happens. It is not thought to be caused by diet, lifestyle or anything the person has done. In inherited prion disease, the person has a mistake (‘mutation’) in the gene that helps make the prion protein. The mutation is passed down from a parent and makes the protein more likely to fold into the wrong shape. In acquired CJD, the harmful prion protein enters the body from outside. This can happen through eating contaminated food, as in variant CJD linked to Bovine Spongiform Encephalopathy, or through certain medical procedures. Once the harmful protein enters the body, it can make the person’s own prion protein misfold.
My family member was diagnosed with CJD caused by a genetic mutation. What does this mean for me?
If someone has an inherited prion disease, close family members may also carry the same genetic mutation. First-degree relatives, such as a child or sibling, carry a 50% risk of having the mutation. A blood test can show whether someone has the mutation. The choice to have this test is a personal one. The result may affect a person’s feelings, family life and future plans. For this reason, people are usually offered genetic counselling before testing. Support is available through the National Prion Clinic for people in the UK. For people elsewhere, local support can be found via the CJD International Support Alliance.
How does CJD affect someone?
CJD affects the brain and nervous system. The symptoms are caused by the harmful prion protein spreading through the brain and causing damage. Symptoms vary from person to person and depend on the type of CJD. Common symptoms include problems with memory and thinking, changes in speech or language, changes in mood, behaviour or personality, problems with balance and movement, and sudden jerking movements of the muscles. These jerks are called myoclonus. As the illness gets worse, people lose the ability to walk and talk and have trouble swallowing. They will also lose control of their bladder and bowels. In the later stages, most people lose awareness of what is happening around them. CJD itself is not thought to cause pain directly. Many people with CJD die from infections, such as pneumonia.
How certain are we in the diagnosis?
It may take some time before CJD is diagnosed. Early symptoms can be vague and may look like more common illnesses. Because CJD is rare, doctors may not suspect it at first. Diagnosis has improved over time. Doctors are more aware of CJD, and specialist tests are now more widely available. In most cases, doctors can be very confident about the diagnosis. They look at the person’s symptoms and examination findings and use tests such as brain scans and spinal fluid tests. Sometimes the diagnosis is less clear. Doctors may need to watch symptoms over time or repeat tests. A post-mortem examination after death can also confirm the diagnosis. Very rarely, doctors may consider a brain biopsy. This is usually only done if they need to rule out another illness that might be treatable.
What is the prognosis?
The outlook depends on the type of CJD. In sporadic CJD, the illness usually gets worse quickly. On average, people live for about 5 months from the start of symptoms, but this varies. Some people decline over a few weeks, while others may live for more than a year. Some types of inherited prion disease progress more slowly over several years.

### Speed and course of the illness

The rapid and often unpredictable tempo of the disease means that families may experience intense emotions such as grief, confusion and helplessness as they struggle to process changes in real time [24, 25, 28]. Anticipatory grief, simultaneously preparing for future loss while coping with ongoing decline, is common [29]. The unexpected onset of the disease can create significant practical challenges, where care requirements might exceed available resources. Families must often quickly learn to manage legal and financial matters, such as securing power of attorney, accessing accounts and navigating end of life decisions, many times without the input from the patient themselves [15, 24, 25]. Professionals can help through proactive care planning discussions and identifying avenues for support.

### Family caregivers

People with prion diseases are often cared for by family members who must quickly adjust to new care roles. Whilst family members taking on caregiving can be beneficial to the patient, as continuity of carers is thought to improve outcomes and assist with orientation, it can place significant stress on the family caregiver, both on their physical and mental health [30–33]. Caregivers of people with dementia have worse health outcomes, report poorer quality of life and are more prone to depression and anxiety [34]. They may change their employment situation to provide care, resulting in financial pressures. Interventions to help support caregivers include sharing disease-specific information about what to expect, training to equip them with the necessary skills, and empowering them to use available resources, such as practical and personal care support, help with domestic chores, mental health support, respite care and financial assistance.

### Knowledge, awareness and stigma

Given the rarity of prion diseases, caregivers are often in the unusual position of having to advocate for and educate others [23]. This can lead to feelings of isolation through lack of peer support and understanding [25]. Misconceptions from the Bovine Spongiform Encephalopathy epidemic have also led to stigma, with inaccurate concerns about transmission creating unnecessary fear. Signposting to trusted websites and peer support networks, such as the CJD Support Network (see Table 1), can be helpful.

### Genetic testing

Genetic testing is routinely offered to people with prion diseases in the UK. This involves obtaining a blood sample from the individual and screening the *PRNP* gene to identify mutations. This adds an additional layer of complexity for families at an already difficult time. In the case of IPD, people may have already lost other family members to prion disease and may be reliving past traumas while also fearing for their own futures. The timing and content of these discussions need to be carefully considered. Support for at-risk family members is available through the National Prion Clinic.

## Infection control

Families and healthcare staff should be advised that there is no evidence to suggest that CJD is transmitted from person to person through regular social and clinical contact. For routine clinical care, including the handling of blood, cerebrospinal fluid and other body fluids, all of which are all low risk for CJD, no additional infection control measures are required beyond what is typically practised for any other patient (e.g. hand hygiene, appropriate use of personal protective equipment, safe handling of sharps and proper cleaning and disinfection) [35].

Rarely, iatrogenic transmission can occur due to certain medical interventions. Procedures involving high-infectivity tissues such as the brain, spinal cord and posterior eye, should be done with additional precautions and instrument management. There is helpful guidance available on minimising transmission risk of prion diseases depending on the nature of the procedure, infectivity of the tissue involved and status of the individual [35].

People with prion diseases, as well as individuals at increased risk of prion disease (due to their family or medical history), are not eligible to be blood or organ donors. They should inform their healthcare team of their risk status prior to undergoing any medical or dental procedures so that the appropriate precautions can be undertaken.

## Symptom management

### Neuropsychiatric symptoms

The neuropsychiatric manifestations of prion diseases are wide-ranging and can include distress, agitation, aggression, disinhibition, hallucinations, delusions, low mood and anxiety. The recognition and management of these symptoms is an important area for patients and carers. In the UK National Prion Monitoring Cohort, a longitudinal observational study, carers reported that ‘behaviour and hallucinations’ was one of the most problematic symptom categories [30].

While guidelines for common dementias, such as Alzheimer’s disease, can be useful for informing management plans, there are important differences between prion diseases and other dementias. For example, prion diseases tend to progress more rapidly towards akinetic mutism. This means that psychiatric symptoms can both emerge and resolve at a relatively rapid rate. Therefore, a watch-and-wait approach to troubling symptoms (e.g. delusional ideas) is often justified.

Assessment and management should be carried out holistically [36], and local guidelines on management of behavioural and psychological symptoms of dementia should be consulted. So-called ‘challenging behaviour’ should generally be seen as the communication of an unmet need, and management should aim to address this need. In cases which do not respond to non-pharmacological interventions, judicious use of psychotropic medication may be required to lessen severe distress (which might look like intolerable agitation, screaming or psychosis that disrupts care and safety) or manage risks to self or others. Examples of significant risks include repeated physical assaults, dangerous wandering, self-injury or severe paranoia leading to the refusal of essential care.

Triggers of behavioural symptoms may be determined through careful observation or by using behaviour charts, such as Antecedent-Behaviour-Consequence charts. People should be assessed for pain, discomfort, dehydration, constipation and physical illness. The individual’s environment, including furniture and light/noise levels, needs to be carefully considered. Movement, touch, bright light and noise can all cause distress, particularly for people with visual impairment and myoclonus. If distress is particularly prominent during delivery of personal care, consider modifications such as more consistency in caregivers (if feasible), more verbal explanations and gentler physical contact.

Based on observations by some authors, true delirium as a reversible acute confusional state appears to be less common in prion diseases, unlike in Alzheimer’s disease or Lewy body dementia. While sudden behavioural or cognitive changes should prompt assessment for infection, medication effects or pain, these symptoms usually represent disease progression rather than a superimposed delirium. Nonetheless, a careful screen for potentially reversible contributors (e.g. constipation, urinary retention, dehydration or drug interactions) is appropriate.

Sleep should be managed through non-pharmacological strategies where possible. These include maintaining a dark, quiet, and comfortable environment and increasing daytime stimulation where possible. Where these approaches are not sufficient, use of a sedating antidepressant, hypnotic or melatonin could be considered and balanced against the risks of falls and daytime sedation.

Symptoms of mood disorder are present in many people with prion diseases at any stage of disease [6], ranging from early depression to emotional lability or apathy in the more advanced stages. Antidepressants may be helpful and are generally well tolerated in that adverse effects are not severe and rarely lead to discontinuation.

Guidance for pharmacological management of neuropsychiatric symptoms is limited. Thompson *et al.* [6] provide some evidence for the use of antipsychotic medication in the management of severe psychotic or agitated features. Benzodiazepines, such as clonazepam, may be appropriate in advanced illness where psychotic and agitated features often coexist with troubling symptoms such as myoclonus or insomnia. Choosing a medication that addresses several symptoms reduces the risks of polypharmacy and tablet burden. In cases where medications need to be given parenterally, our preference is for benzodiazepines such as lorazepam or midazolam due to the potential for extrapyramidal side effects from haloperidol to worsen hypertonia. Based on small numbers, acetylcholinesterase inhibitors show promise for the treatment of psychotic symptoms [6, 37]. Table 3 shows suggested approaches based on current expert practice and informed by existing guidelines for more common dementias. Dose ranges for medication are taken from the Maudsley Prescribing Guidelines [38] and are generally those recommended for older adults.

**Table 3:** Medications to be considered in the management of neuropsychiatric symptoms in prion diseases, based on evidence in other neurodegenerative conditions

Symptom	Agent	Route	Usual starting dose	Usual maintenance dose	Usual maximum dose	Comments
**Agitation and aggression**	Antipsychotics					Also used for psychosis with the same dose ranges for olanzapine and quetiapine and double the dose range for risperidone.
	Risperidone (1st line)	Oral	0.25 mg daily or twice daily (BD)	0.5 mg BD	2 mg daily	Risperidone is indicated for the short-term treatment (up to 6 weeks) of persistent aggression in people with moderate to severe Alzheimer’s dementia unresponsive to non-pharmacological approaches and when there is a risk of harm to self or others. There is some efficacy described for anxiety, agitation, psychosis and behaviour management in prion diseases [39].
	Olanzapine	Oral	2.5 mg nocte	2.5–10 mg daily	10 mg nocte	Olanzapine has some positive efficacy data for reducing aggression in Alzheimer’s dementia [40]. There are some positive reports of efficacy described for psychosis and behaviour management in prion diseases [39].
	Quetiapine	Oral	12.5–25 mg daily	50–100 mg daily	100–300 mg daily	Quetiapine has failed to show effectiveness for behavioural and psychological symptoms of dementia except at higher doses (100–200 mg/day) [38]. However, it has a low propensity for movement disorders.
	Benzodiazepines					Consider falls risk in any ambulatory individual. Benzodiazepines have some positive efficacy described for anxiety, agitation, insomnia and behaviour management in prion diseases [39].
	Lorazepam	Oral	0.5 mg daily	0.5–2 mg daily	2 mg/day	
	Diazepam	Oral	1 mg three times daily (TDS)	1 mg TDS	7.5–15 mg/day in divided doses (for anxiety)	
	Clonazepam	Oral	0.5 mg daily	1–2 mg/day	4 mg/day	Clonazepam is commonly used to treat myoclonus.
**Low mood/anxiety**	Sertraline (or alternative selective serotonin reuptake inhibitor (SSRI))	Oral	25–50 mg mane (25 mg can be increased to 50 mg after one week)	50–100 mg mane	100 mg (occasionally up to 150 mg mane)	SSRIs have mixed reports of efficacy for treating mood disturbance in prion diseases [39].
	Mirtazapine	Oral	7.5 mg nocte or 15 mg nocte	15–30 mg nocte	45 mg nocte	Lower doses (7.5–15 mg) may be useful for treatment of insomnia due to preferential antihistaminergic activity at lower plasma concentrations. The sedating effect is reduced at higher doses. There is some efficacy described for mood disturbance in prion diseases and it is well tolerated [39].
**Visual hallucinations**	Donepezil	Oral	5 mg daily	10 mg daily		
	Rivastigmine	Oral	1.5 mg BD	3–6 mg BD		
		Patch	4.6 mg/24 h	9.5 mg/24 h		
**Insomnia**	Zopiclone	Oral	3.75 mg nocte	3.75–7.5 mg nocte	7.5 mg nocte	Consider falls risk in any ambulatory individual. There are some positive data for use of melatonin and benzodiazepines for abnormal sleep in prion diseases [39].
	Zolpidem	Oral	5 mg nocte	5 mg nocte	5 mg nocte	
	Melatonin	Oral	2 mg (modified release) once daily (1–2 h before bedtime)	2–10 mg (modified release) once daily (1–2 h before bedtime)	10 mg (modified release) once daily (1–2 h before bedtime)	
	Trazodone	Oral	50 mg nocte	50–100 mg nocte	150 mg nocte	
	Clonazepam (or alternative benzodiazepine)	Oral	0.25 mg nocte	0.25–1 mg nocte	1 mg nocte	

In extreme cases, where there is an immediate and severe threat to safety of the individual or others, detaining the patient under the relevant legal framework (e.g. the Mental Health Act in the UK) may be necessary, which can enable rapid escalation of medications in a closely monitored setting.

### Motor symptoms

Prion diseases cause a range of motor symptoms, from the more common gait ataxia, myoclonus and hypertonia to the less common tremor, chorea and alien limb phenomenon. Not all are amenable to treatment; for example, there are no proposed pharmacological treatments for ataxia. Where treatments exist, the evidence base is very limited according to a 2024 scoping review [39]. In the management of all motor symptoms, we recommend involving the MDT, particularly in the early stages of disease when there are opportunities for disability to be minimised. Occupational therapy is especially helpful for advising on mobility aids, home adaptations and adaptive equipment for activities of daily living. It is also important to review medications which increase falls risk or worsen motor symptoms. Consider also any pre-existing comorbidities which may contribute to mobility issues.

Myoclonus, characterised by brief, shock-like involuntary muscle contractions, is a hallmark feature particularly of sporadic CJD. Myoclonus can be spontaneous (without trigger), action-induced (on movement) and stimulus-sensitive (to light, sound or touch). Where possible, the environment should be optimised and triggering stimuli avoided or limited. Infrequent myoclonus may not necessarily require intervention, whereas someone whose myoclonus interferes with their daily functioning or care and is distressing should be offered treatment. Myoclonus may also be distressing for families to witness; this should be addressed by explanation and reassurance before considering drug treatment.

Levetiracetam and clonazepam are commonly used medications for myoclonus, with some described efficacy in the literature [7, 39]. Levetiracetam is generally well tolerated, easy to titrate and available in multiple formulations. We recommend reserving clonazepam for the more advanced stages, when people are no longer ambulant, due to the adverse effects of benzodiazepines on cognition, coordination and falls risk. Medications related to levetiracetam (such as piracetam or brivaracetam), or sodium valproate can be trialled if there are contraindications to first-line medications. At the end of life, medications for myoclonus can be transitioned to levetiracetam or midazolam via continuous subcutaneous infusion. In severe cases, use of multiple agents may be required. If a medication results in adverse effects, avoid abrupt withdrawal if possible. It is also worth noting that some medications can cause or worsen myoclonus, including opioids, antidepressants, antipsychotics and some antiepileptics (such as phenytoin, carbamazepine, lamotrigine and gabapentinoids) [41].

Increased tone in prion diseases can manifest as rigidity, spasticity or paratonia (involuntary resistance to passive movement). This can sometimes be difficult to clinically characterise, which complicates choice of treatment. Medications which cause extrapyramidal side effects, such as typical antipsychotics, should be used with caution, especially in the early stages when people still retain a higher level of function. Effective pharmacological interventions for increased tone are limited, though there are some reports suggesting efficacy of baclofen for spasticity and levodopa for rigidity [39]. It is often most severe in the advanced stages of disease [7], when people are akinetic and bedbound, and treatment is unlikely to have an impact on disability. Given a lack of effective drugs, we often do not directly treat hypertonia at this stage and instead focus on optimising nursing care, preventing pressure injury and relieving pain. Passive range of motion and massage are good opportunities to involve families in patient care.

There are scarce data on the management of tremor in prion diseases, but it would be reasonable to try propranolol if treatment is indicated. Other hyperkinetic movement disorders such as chorea, alien limb phenomenon and dystonia are less common and may resolve as people approach akinetic mutism [7]. Evidence for drug efficacy is limited to case reports. One paper reported some benefit in using haloperidol for chorea and dystonia [42]. Given that symptoms can evolve rapidly, medications can be trialled with frequent monitoring for clinical efficacy, adverse effects and ongoing need for treatment, with a low threshold to discontinue. Table 4 summarises medications considered by experts, informed by the limited evidence base as discussed.

**Table 4:** Medications used for the management of motor symptoms in prion diseases

Symptom	Agent	Route	Usual starting dose	Usual maintenance dose	Usual maximum dose	Comments
Myoclonus	Levetiracetam (1st line)	Oral (PO); intravenous (IV); continuous subcutaneous infusion (CSCI)	250–500 mg BD	500–750 mg BD	1.5 g BD	First-line treatment in ambulatory people. Available in PO (tablets, granules, oral solution) and IV preparations. The total daily dose can be given over 24 h via continuous subcutaneous infusion at end of life.
	Clonazepam (1st line)	PO	0.25–0.5 mg nocte	0.25–1 mg BD	4–8 mg per day (may be given in 2–4 divided doses)	Use with caution in ambulatory people. Clonazepam is also used for anxiety and tremor. It has a longer duration of action compared with other benzodiazepines. It may produce dependence and/or tolerance.
	Midazolam (end of life)	CSCI	10–20 mg/24 h	10–60 mg/24 h	60 mg/24 h	
	Sodium valproate	PO; IV	200–500 mg daily (in 1–2 divided doses, depending on formulation)	1–2 g daily (in 2–3 divided doses)	2.5 g per day (in 2–3 divided doses)	Available in PO (tablets, granules, capsules, oral solution) and IV preparations.
	Piracetam	PO	7.2 g daily (in 2–3 divided doses)	7.2–12 g daily (in 2–3 divided doses)	24 g per day (in 2–3 divided doses)	Only available as tablets.
	Brivaracetam	PO; IV	25–50 mg BD	25–100 mg BD	100 mg BD	Available in PO (tablets, oral solution) and IV preparations.
**Rigidity**	Levodopa	PO	25/100 mg TDS	25/250 mg TDS	200/2000 mg per day (in divided doses)	
**Spasticity**	Baclofen (1st line)	PO	5 mg TDS	5–20 mg TDS	100 mg per day	
	Tizanidine	PO	2 mg daily	2–24 mg daily (in 1–4 divided doses)	36 mg per day	
	Diazepam	PO	2–5 mg daily	2–15 mg daily (in divided doses)	60 mg per day	
	Dantrolene	PO	25 mg daily	75 mg TDS	100 mg QDS	
**Tremor**	Propranolol	PO	40 mg BD or TDS	80–160 mg daily (in divided doses)	160 mg daily (in divided doses)	
	Primidone	PO	50 mg daily	50–250 mg TDS	250 mg TDS	

### Seizures

Seizures are uncommon, occurring in around 10% of sporadic CJD cases, and less frequently in other prion diseases, with the exception of IPD due to E200K mutation [43]. When seizures occur, they are usually during the course of the disease and rarely occur at presentation. The evidence base for management of seizures in prion diseases is limited to case reports and series and suggests that most cases do not respond to anti-seizure medications [44]. If treatment is needed, we recommend levetiracetam as first-line due to its tolerability, broad spectrum of action and efficacy against myoclonus, thus treating two symptoms. Similarly, benzodiazepines are effective for both seizures and myoclonus.

### Pain

Pain is generally not found to be a problematic symptom in sporadic CJD [30], with the exception of distal pain sometimes seen in the thalamic presentation (2% of sporadic CJD cases) [5], but it should not be dismissed when present. Given the lack of research into pain in prion diseases, here we borrow principles of pain assessment and management from the wider dementia literature.

A comprehensive pain assessment is often limited by the person’s cognitive impairment and as such, collateral history from caregivers is invaluable. Pain in the non-verbal individual may manifest as agitation or restlessness. Structured observational assessment tools such as the PAINAD scale [45], DisDAT [46], or the Abbey pain scale [47], while not validated for use in prion diseases, may be helpful to supplement a standard clinical assessment.

Underlying causes of pain, such as constipation or pressure injuries, should be treated directly. Frequent repositioning can help with pain related to immobility. A stepped approach for pharmacological management, with regular paracetamol as first-line, should be considered [48]. Opioids have adverse effects on cognition and falls risk and therefore should be used with caution, under the guidance of palliative care specialists. Upon initiation of any intervention, the person should be reviewed regularly to assess effectiveness.

Pain, particularly in the form of dysaesthesia, is sometimes seen in acquired [49, 50] and inherited prion diseases [51]. In our practice, we find the first-line agents used for neuropathic pain (amitriptyline, duloxetine, gabapentin and pregabalin) in standard doses to be effective, although several drugs may need to be tried successively. Carbamazepine may also be helpful in treating bothersome sensory symptoms in acquired CJD [52].

### Saliva management

Excessive saliva can result from either overproduction (sialorrhoea) or reduced clearance due to changes in swallow. Overproduction is commonly caused by local infections (such as fungal infections or gum disease), or stimulation from a foreign body (such as a nasogastric tube). Reduced clearance occurs when the frequency of swallowing decreases or the swallow itself becomes impaired, which carries a possible aspiration risk.

Management of excessive saliva begins with good oral care and dental hygiene, alongside a review of medications. Addressing reversible causes, such as local infections, is an important early step. In some cases, head positioning or gentle anterior oral suctioning may alleviate saliva buildup. Local palliative care guidelines on excessive salivation should be followed. Trials of anticholinergic medications such as glycopyrronium bromide or hyoscine hydrobromide can be considered, but they should be used with caution as they may cause thicker, stickier saliva as well as worsening cognitive impairment if used systemically.

Reduced saliva or dry mouth can result from mouth breathing or side effects of medications (e.g. anticholinergic agents). Approaches to management include encouraging hydration (although this may increase aspiration risk in those with an impaired swallow), maintaining good oral care and artificial saliva sprays.

### Nutrition and hydration

There is a naturally strong wish to nurture a person through their illness, which is usually associated with the provision of food and drink. For much of the illness, this approach is to be encouraged. However, swallowing will deteriorate over time due to progressive physical and cognitive changes, giving rise to the risk that oral feeding will become actively harmful due to aspiration. Cognitive impairment may also manifest as refusal of food or drink, especially from unfamiliar people and if the person is being hand-fed.

Risks of harm should be balanced against the benefits to the person of receiving food and drink by mouth and the psychological harm, if any, of not doing so. Conversely, there may be some people who wish to refuse nutrition and hydration, or for whom the withdrawal of, or decision not to provide, CANH (which includes the use of all forms of tube feeding), in a person’s best interests, will hasten death. This is a complex area of law and we direct readers to national and professional guidance [53, 54] on these matters.

A review of the individual’s capacity and FCP documents should be considered prior to initiating a best interests process. A multidisciplinary discussion to inform best interests decision-making should consider the person’s clinical status, prognosis, wishes and the views of family. If there is unresolvable conflict among healthcare professionals and the person’s family, legal advice can be sought, although this may be difficult to access in the accelerated time frames of prion disease.

If the decision is made to proceed with eating and drinking with acknowledged risks, it is important to consider methods of risk reduction. These include involvement of speech and language therapy for specialist assessment and expertise, dietary modifications, good oral and dental care and documentation of a clear care plan so that family and staff are aligned. Note that there is probably a risk of aspiration (and aspiration-related death) due to saliva alone, and risk also reflects non-modifiable factors such as age, frailty and comorbidities. In this context, it may be that the additional risk due to the provision of food and drink is considered small.

Research into the provision of CANH through a nasogastric or gastrostomy tube shows that the additional survival time associated with this approach is found in a prolongation of the akinetic-mute period [55, 56]. Whether it is in the best interests of a person in a state of akinetic mutism to receive CANH is a decision to be made on an individual basis. Current national guidance states that CANH should not routinely be used in people living with severe dementia, unless indicated for a potentially reversible comorbidity [57]. In the rare instances of prion disease where it is deemed in the person’s best interests to receive CANH, the decision should be reassessed regularly as part of every care review. This allows for adjustments to be made in response to changes in the individual’s clinical condition, such as inability to tolerate the intervention, changes in level of alertness, complications such as infections or entering the actively dying process. If there is agreement that CANH should be withdrawn and professional guidance has been followed, CANH should be discontinued as soon as is reasonably practicable, with a detailed plan in place for the process of withdrawal and end-of-life care [53].

A small number of IPD patients with PrP truncation mutations (PrP systemic amyloidosis) experience prolonged gastrointestinal disturbance with diarrhoea, caused by autonomic neuropathy and extensive amyloid deposition in tissues, especially the gut. This often leads to weight loss. In this instance, following attempts to manage with oral medications such as loperamide, total parenteral nutrition may be considered for nutritional support. Other forms of IPD (notably A117V) are associated with early pseudobulbar palsy and dysphagia, prior to significant cognitive involvement. In these cases, the use of CANH is more clearly justifiable.

### Incontinence

Continence issues evolve as the disease progresses, from needing prompting and assistance with hygiene in the early stages to double incontinence in the later stages. A continence assessment should consider factors such as dietary habits, functional impairment, disease-specific effects (such as autonomic dysfunction in IPD) and medication side effects.

Non-pharmacological interventions include scheduled toileting, clothing modifications, environmental adjustments and continence pads. Referral to local continence services is recommended. Occupational therapy involvement is also important for home adaptations and specialised equipment. Regular skin assessments are crucial to prevent complications associated with incontinence, with referral to local tissue viability services if needed.

Pharmacological therapies are rarely used due to limited efficacy but can be considered in select people with particularly bothersome symptoms. Anticholinergic medications (solifenacin, tolterodine or trospium) or beta-3-receptor agonists (mirabegron) are used for symptoms of overactive bladder, while alpha blockers (tamsulosin) are preferred for voiding dysfunction [58]. Adverse effects may be problematic (for example, worsening cognition with anticholinergic medications) and should be closely monitored. It should also be noted that cholinesterase inhibitors, which are used for neuropsychiatric symptoms of prion diseases such as visual hallucinations, can cause bladder overactivity. In the advanced stages, where severe rigidity or tissue breakdown make frequent nursing care distressing or challenging, external or indwelling catheters should be considered [31].

### End of life care

Towards the end of life, people with prion diseases are akinetic, mute and require full nursing care. Data from the National Prion Monitoring Cohort study show that in the UK, 28% of people with prion disease die at home, 28% in hospice, 23% in a residential or nursing home and 21% in hospital. By contrast, among people with dementia, only 12.1% die in hospital and 3.2% in hospice [59]. The higher proportion of hospital and hospice deaths in CJD highlights the complex care needs associated with the disease. Due to the rapid course, people may not have had an opportunity to discuss their preferences for care. FCP documents should be reviewed and respected. Healthcare staff should undertake conversations with the person’s family to understand their views, values and preferences.

Informed by FCP, medications and interventions should be reviewed and discontinued unless essential to care at the end of life. The healthcare team should document a treatment plan, including escalation plans (e.g. rationale for considering intravenous antibiotics).

Symptom assessment is challenging when people are unable to communicate, the disease is rapidly progressing and symptoms evolving. Symptom prevalence studies in people dying with prion disease are not described in the literature. Proxy-completed assessment tools, such as the IPOS-Dem [60] or the previously mentioned pain scales may be helpful, although their evidence base is gathered in slowly progressive neurodegenerative disease and learning disability, and there is a paucity of evidence for their use in prion diseases.

As with many aspects of caring for people with prion diseases, symptom management at the end of life requires a multidisciplinary approach. Nursing care should focus on the maintenance of skin and corneal integrity, mouth care and management of incontinence and upper airways secretions. Physiotherapy and occupational therapy input is helpful in supporting caregivers to move the person and advising on the management of contractures and other motor symptoms. Other team members, such as music therapists and chaplains, can help support the person’s and family’s psychological, spiritual, religious and cultural needs.

The healthcare team should prescribe anticipatory medications so nursing staff can manage symptoms without delay. People who are dying are unable to swallow oral medications safely, therefore a subcutaneous or intravenous route is commonly used. Local palliative care prescribing guidelines informed by NICE guidance [61] are a good starting resource to address common end-of-life symptoms, including excessive upper airways secretions and agitation. Medications used for myoclonus can be transitioned to continuous subcutaneous infusion of midazolam or levetiracetam (see Table 4).

## Post-death care

The development and routine clinical use of MRI brain and CSF RT-QuIC in prion diseases have improved diagnostic confidence in life and reduce the need for post-mortem examinations. There are instances where the individual, family or physician may pursue a consented hospital post-mortem examination, to:

- Confirm or exclude a diagnosis of prion disease, in cases of clinical uncertainty
- Establish the aetiology and sub-classification of prion disease
- Acquire tissue to test for pathogenic variants of the *PRNP* gene, when blood could not be taken in life
- Follow the wishes of the family for a definitive diagnosis
- Donate tissue for prion research

In arranging a post-mortem, the physician should seek advice and discuss requirements with their local pathologist. A ‘high-risk’ post-mortem suite is desirable, but any general hospital post-mortem suite may be used with the addition of some important health and safety measures [35]. In most cases, a consented post-mortem examination is limited to the brain. Full neuropathological examination requires sampling from many specified regions across the brain. Sampling after fixation increases the likelihood of reaching a diagnosis, so ideally the consent should seek retention of the brain for prolonged fixation. Following completion of the examination, the brain can be returned, respectfully disposed of or donated for research or education as detailed in the consent. Alternatively, sampling can be done from the fresh brain at the time of post-mortem, which allows for only necessary samples to be retained and the opportunity for the remainder of the brain to return to the body prior to funeral without delay [62]. Analysis of post-mortem brain samples from suspected cases of prion disease should be handled in a microbiological containment level 3 laboratory (with derogations) until formalin fixed and formic acid-treated [63]. These extensive investigations can take up to 3 months. Families who wish to view or touch the deceased should not be discouraged. Preferably this contact should occur prior to the post-mortem examination, as the person may look different afterwards, which can be distressing to families.

## Bereavement care

Both pre- and post-death, advice and help are available from national CJD specialists. Support can also be found locally from the GP, palliative care team and hospital bereavement services, as well as third sector organisations, such as the CJD Support Network. Additional resources are included in Table 1. In cases of IPD, follow-on care can be provided for at-risk family members by the National Prion Clinic.

## International perspective

Palliative and supportive care for CJD shares core clinical aims across health systems, namely timely recognition of rapid functional decline, proactive communication about prognosis and uncertainty, early advance care planning and skilled symptom management that prioritises comfort, dignity and family support. However, despite sizable efforts led by the World Health Organization since the 1980s, there remain significant insufficiencies in global palliative care approaches, which are well described in international mapping exercises [64]. Many nations have yet to frame palliative care within their priorities for health, or where there are efforts, they may not be well integrated and thus limited in their impact.

Differences in how palliative care for CJD is approached internationally arise from the organisation and funding of healthcare, the availability of specialist services and cultural expectations around end-of-life decision making, particularly regarding clinically assisted nutrition and hydration. The UK model described in this manuscript sits within a tax-funded system with care that is largely free at the point of delivery, a comparatively centralised national specialist infrastructure for prion disease, and additional disease-specific resources that can be mobilised quickly. In contrast, the approaches in Japan, Germany and the USA illustrate how the same clinical problems are managed within distinct cultural and structural constraints.

### Japan

In Japan, the diagnostic process for suspected CJD is broadly similar to that in the UK, but there are important differences in palliative care practice, particularly around nutrition and the usual place of care at the end of life. Japan has established national prion disease clinical practice guidance, with an updated guideline scheduled for publication in 2026, widely used by neurologists and includes palliative care recommendations such as symptomatic management of motor and psychiatric symptoms, rehabilitation-oriented approaches and nutritional planning [65]. The most prominent divergence from UK practice relates to decision-making about assisted nutrition in the context of dysphagia. Japanese practice is strongly influenced by family preferences and wider social norms, in which declining nutritional support when swallowing becomes unsafe can be difficult, especially where there is no clear contemporaneous statement of the person’s wishes. This can be reinforced by broader societal expectations about maintaining nutrition in advanced illness and, in older adults, the perceived moral weight of preventing ‘starvation’ even when the underlying disease is irreversible [66].

As a consequence, tube feeding is common for people with CJD in Japan, including in the terminal phase of disease. Observational data suggest that enteral feeding is associated with longer survival in Japanese sporadic CJD cohorts [56], and international comparisons have reported longer duration of prion disease in Japan than in other countries [67]. These patterns are clinically consequential. Longer survival may extend time spent in profound disability, shifting caregiver burden, resource utilisation and the duration of complex symptom management needs. They also shape where people die. In Japan, the high prevalence of tube feeding and the logistical complexity of managing nutrition and aspiration risk contribute to deaths occurring predominantly in hospitals, including long-term care facilities, with home deaths comparatively uncommon.

### Germany

Germany’s supportive and palliative care pathways for CJD are broadly aligned with UK principles, including the value placed on early coordination of community support, involvement of palliative care expertise and practical planning for fast deterioration. Home nursing is, in principle, available through statutory health insurance, but the speed of implementation may be constrained by administrative processes and variable familiarity with prion disease among assessors. As a result, initiation of intensive support can be delayed relative to clinical need, particularly if referral to social work or care coordination occurs late in the diagnostic admission. In some regions, hospital-based social work teams can accelerate arrangements, but this depends on local practice and on active engagement by families at a time of high distress.

A distinctive feature of the German context is the potential role of the national surveillance unit in facilitating access to services. Where insurance approvals or hospice admissions are hindered by uncertainty about prognosis or perceived ambiguity in diagnosis, brief specialist clinical statements about expected disease course can be helpful in overcoming administrative barriers. Specialist inpatient palliative care units exist in some centres, and specialist outpatient palliative care and hospice services are available in many regions, but coverage remains variable and is not uniformly accessible. This creates an important parallel with the UK, where early identification of local palliative resources and realistic planning for escalation of support are central, but with an additional layer of insurance-dependent variability.

Germany also illustrates how funding and reimbursement pathways can influence diagnostic and counselling processes. In some settings, reimbursement for *PRNP* genetic testing can be administratively complex, and inpatient testing may be difficult to fund. Where outpatient reimbursement is restricted to certain recognised specialists, this can delay genetic clarification and inadvertently create additional burdens for families, particularly if testing is deferred until after discharge. This matters for palliative care because genetic confirmation can influence family counselling, risk communication and practical planning. Finally, approaches to gastrostomy have evolved. Whereas percutaneous endoscopic gastrostomy was reportedly more common historically, more recent practice has seen a marked decline, with gastrostomy now used only in exceptional cases. In Germany, most people die in palliative care institutions, including hospital palliative wards, nursing homes or hospices, with smaller proportions dying at home or during acute diagnostic admissions.

### United States

In the USA, the need for palliative care in prion disease is amplified by rapid progression, but access and timing are shaped by fragmented healthcare delivery and insurance-dependent eligibility. In contrast to the UK’s comparatively centralised pathways and nationally organised specialist services, US care is often dispersed across multiple providers and settings, which can delay diagnosis, complicate coordination and postpone palliative care referral. Real-world evidence suggests a high healthcare burden and a compressed clinical course; the average time from symptom onset to death has been reported as ~7.9 months, with a large proportion of people dying within 2 months of diagnosis [68]. This combination of rapid decline and diagnostic complexity makes early palliative engagement particularly important, but also logistically challenging.

Despite the universally fatal prognosis, palliative care appears underutilised. A recent retrospective study reported that only around two-thirds of US prion disease patients accessed palliative care, with referrals often occurring late in the disease course [69]. The reasons are likely multifactorial, including late recognition of prognosis, the misconception that palliative care is synonymous with imminent dying, and differences in insurance coverage that affect service availability. Unlike the UK, there are no disease-specific national care funds or routinely available specialist nursing services for prion disease. Support is instead commonly provided through non-profit and federal infrastructures. Families may access education and peer support through organisations such as the CJD Foundation, and definitive diagnosis and subtyping via autopsy can be facilitated by the US National Prion Disease Pathology Surveillance Center, funded by the Centers for Disease Control and Prevention. These elements provide important support but do not substitute for consistently accessible, early, multidisciplinary palliative care embedded within routine clinical pathways.

Taken together, the international differences are not in patient need, but in how health systems and cultural norms determine what is feasible and what is expected. At the service level, the comparisons emphasise the importance of rapid mobilisation of community nursing, hospice and specialist palliative support, and the role of national prion services in advocacy, education and coordination. International variation also suggests that outcomes such as place of death and duration of advanced disability are partly modifiable and may be shaped by policy decisions, not only by disease biology.

## Conclusion

Prion diseases present a distinct and urgent set of challenges for healthcare professionals, patients and families alike. Their rapid progression, complex symptomatology and profound psychosocial impact demand an adaptive, compassionate and evidence-informed approach to care. While no proven disease-modifying therapies currently exist, the thoughtful application of palliative principles—combined with strong multidisciplinary collaboration and system-wide support—can improve quality of life and uphold patient dignity. We hope this review, unique in bringing together the expertise of a broad range of multidisciplinary specialists, serves as a practical resource for healthcare professionals navigating the complexities of prion diseases, and as a foundation for future research.

## Supplementary Material

Supplementary_materials_afag206
