# Supplementary material for: Multidisciplinary recommendations for palliative and supportive care in Creutzfeldt-Jakob disease and related disorders
Source: Age Ageing. 2026 Jul 19;55(7):afag206. doi: 10.1093/ageing/afag206 (PMC13381037; doi:10.1093/ageing/afag206)
Supplement: Supplementary_materials_afag206 [file supplementary_materials_afag206.zip › Supplementary_materials_afag206.docx]

**Multidisciplinary Recommendations for Palliative and Supportive Care in Creutzfeldt-Jakob Disease and Related Disorders**

**Appendix 1: case study.**

Mrs X, a 75-year-old woman, presented to the emergency department and was admitted under care of the elderly with a two-month history of memory impairment and personality changes. She also started having falls and needing a cane for balance difficulties. Examination revealed cerebellar ataxia, rigidity, dystonic posturing of the left arm, and myoclonus. Non-pharmacological interventions, including distraction and gentle reassurance, were mostly successful at managing behavioural difficulties such as wandering and taking other people’s belongings. However, there were episodes of agitation and aggressive behaviour despite these measures and atypical antipsychotics were added with partial effectiveness.

She was reviewed by neurology who had a strong suspicion of CJD based on the clinical features and supportive MRI findings. Mrs X was promptly referred to national CJD services who confirmed the diagnosis, and together with the hospital palliative care team, supported the patient, family, and ward team with symptom management, psychological support, future care planning, and discharge planning. There had been concerns surrounding hypersomnolence with using lorazepam for managing difficult behaviour previously, but it was reinstated successfully on an as required basis. Emotional and pre-bereavement support was given to Mrs X’s family.

Mrs X could not retain information about her condition and was felt not to have capacity to make decisions about place of care, so the palliative care team consulted with her family to make a decision based on her best interests. Her family reported that she had always disliked hospitals. Considering this preference and her clinical needs, she was discharged to the local hospice five days later. In the hospice, her myoclonus worsened which made care challenging and was felt to be distressing. Clonazepam was used with success. This was eventually switched to midazolam via continuous subcutaneous infusion when it was felt she was reaching end of life, and a personalised care plan for the last days of life commenced. She died peacefully ten days after admission to hospice. Final confirmation of her diagnosis through a positive RT-QuIC result was not received until after discharge to the hospice, and at that point she would not have been well enough for discharge from hospital, highlighting the importance of timely referral to national specialists and palliative care.
